# Can Generative Artificial Intelligence Enhance Health Literacy About Lateral Epicondylitis?

**DOI:** 10.7759/cureus.61384

**Published:** 2024-05-30

**Authors:** Michael J Miskiewicz, Christian Leonardo, Salvatore Capotosto, Kenny Ling, Dorian Cohen, David Komatsu, Edward D Wang

**Affiliations:** 1 Department of Orthopaedic Surgery, Stony Brook University, Stony Brook, USA; 2 Department of Orthopaedics, Stony Brook University Hospital, Stony Brook, USA

**Keywords:** health literacy, patient education materials, chatgpt, lateral epicondylitis, orthopedic surgery

## Abstract

Introduction: Health literacy is a critical determinant of a patient’s overall health status, and studies have demonstrated a consistent link between poor health literacy and negative health outcomes. The Centers for Disease Control and Prevention (CDC) and the National Institutes of Health (NIH) advise that patient educational materials (PEMs) should be written at an eighth-grade reading level or lower, matching the average reading level of adult Americans. The purpose of this study was to investigate the ability of generative artificial intelligence (AI) to edit PEMs from orthopaedic institutions to meet the CDC and NIH guidelines.

Methods: PEMs about lateral epicondylitis (LE) from the top 25 ranked orthopaedic institutions from the 2022 U.S. News & World Report Best Hospitals Specialty Ranking were gathered. ChatGPT Plus (version 4.0) was then instructed to rewrite PEMs on LE from these institutions to comply with CDC and NIH-recommended guidelines. Readability scores were calculated for the original and rewritten PEMs, and paired t-tests were used to determine statistical significance.

Results: Analysis of the original and edited PEMs about LE revealed significant reductions in reading grade level and word count of 3.70 ± 1.84 (p<0.001) and 346.72 ± 364.63 (p<0.001), respectively.

Conclusion: Our study demonstrated generative AI’s ability to rewrite PEM about LE at a reading comprehension level that conforms to the CDC and NIH guidelines. Hospital administrators and orthopaedic surgeons should consider the findings of this study and the potential utility of artificial intelligence when crafting PEMs of their own.

## Introduction

Health literacy, or the ability of patients to understand health-related information, stands as one of the most significant indicators of an individual's overall health [1]. Past research has consistently linked low health literacy to negative health outcomes [2-3]. The surge in global internet usage by over 11-fold in recent years [3] presents healthcare providers with a new opportunity to disseminate information to their patients. This shift towards online patient education necessitates an awareness of the average patient's reading comprehension abilities by physicians to ensure the highest quality of patient education material (PEM).

Data from the National Center for Education Statistics indicate that in the United States, the average person reads at about the level of an eighth grader [4]. Considering this, both the Centers for Disease Control and Prevention (CDC) and the National Institute of Health (NIH) advise that PEMs should be written at no higher than an eighth-grade reading level [5-6]. However, research indicates that many hospital and physician websites offer PEMs at reading levels that far exceed these recommendations [7-10]. This situation underscores the pressing need for PEMs to be revised to a level accessible and understandable to the general population.

Lateral epicondylitis (LE), commonly known as “tennis elbow,” represents a form of tendinosis of the extensor muscles of the forearm and is one of the most common sources of lateral elbow pain [11]. This condition has traditionally been linked to physical activities that require repetitive motion of the arm and wrist, such as playing tennis or using a screwdriver at work [12]. Consequently, blue-collar workers whose jobs predominantly involve manual labour may be at particularly high risk for developing LE [11-13]. This raises concerns about the accessibility and effectiveness of PEMs targeted to individuals suffering from LE.

One potential solution to this discrepancy between the CDC’s recommendations and the actual readability of available online PEMs involves utilising artificial intelligence. In response to the skyrocketing global interest in artificial intelligence (AI), many companies are exploring innovative ways to introduce AI-based technology into everyday life. One notable example of such companies is OpenAI with its widely used online chatbot, Chat Generative Pretrained Transformer (ChatGPT). ChatGPT is a natural language processing tool that engages users in a conversational dialogue [14]. While the full versatility of ChatGPT is still being explored, it attracts over one hundred million weekly users who utilise it for professional, educational, and recreational purposes [14-16].

The goal of this study is to explore the ability of ChatGPT to generate PEMs on LE that are comprehensible to most Americans, while considering the average national reading grade level. We hypothesise that by incorporating recommendations proposed by the CDC and NIH, ChatGPT will be able to improve the readability of these PEMs in efforts to bridge the gap in health literacy between patient and provider.

## Materials and methods

The top 25 orthopaedic institutions, as ranked by the 2022 U.S. News & World Report Best Hospitals Specialty Ranking, were selected for this study. The websites of these institutions were searched for PEMs pertaining to LE. The content from each website was copied and preserved for analysis, excluding any audiovisual multimedia such as pictures, diagrams, and videos. Institutions lacking relevant PEMs on LE were excluded from the study.

Each institution’s PEMs were individually uploaded to ChatGPT Plus (version 4.0). ChatGPT was prompted with a message to rewrite the PEM with the following parameters: (1) limit the total number of polysyllabic words to less than 30, (2) limit sentences to less than 10 words, (3) limit paragraphs to less than five sentences, (4) eliminate as much medical jargon without compromising accuracy, (5) when eliminating medical jargon is not possible, provide a brief explanation of the relevant concept, and (6) overall, rewrite this as if you were speaking to an eighth grader. These parameters were based on pertinent recommendations set forth by both the CDC’s Simply Put and NIH Clear & Simple documents [5-6]. A senior orthopaedic surgery resident reviewed all rewritten PEMs to verify the accuracy of the information presented.

Readability scores for both the original PEMs and those rewritten by ChatGPT were calculated using the Readability Formulas website [17]. For each set of PEMs, readability was assessed using seven distinct formulas: Gunning Fog, Flesch-Kincaid Grade Level, Coleman-Liau Index, Simple Measure of Gobbledygook (SMOG) Index, Automated Readability Index, Linsear Write Formula, and FORECAST Readability Formula. These formulas evaluate different elements of text composition, including total word count, the presence of polysyllabic words, and overall language complexity, to derive a readability score, which is indicative of the text's grade-level comprehension requirement (Table 1). The study calculated the average readability score and word count for the PEMs of each institution. To assess the significance of the differences between the original and ChatGPT-revised PEMs, paired t-tests were conducted. Statistical analyses were performed using SPSS software (Version 29.0.0(241); Armonk, NY: IBM Corp), with a p-value of 0.05 or lower indicating statistical significance.

**Table 1:** Description of readability tools and their corresponding formulas.

Readability Tool	Formula
Gunning Fog	Grade level = 0.4 (average sentence length/percentage of hard words)
Flesch-Kincaid Grade Level	Grade level = (0.39 x average sentence length) + (11.8 x average # syllable per word) - 15.59
The Coleman-Liau Index	Grade level = 0.0855 (average # of letters per 100 words) - 0.296 (average # of sentences per 100 words) - 15.8
SMOG Index	Grade level = 3 x square root of polysyllable count
Automated Readability Index	Grade level = 4.71 (characters/words) + 0.5 (words/sentences) - 21.43
Linsear Write Formula	n = [(2 syllable words x 1) + (3 or more syllable words x 3)]/number of sentences If n <20, Grade level = n/20 If n>20, Grade level = n-2/20
FORCAST Readability Formula	Grade level = 20 - (# of single syllable words x 150 / # of words x 10 )

## Results

**Figure 1:**
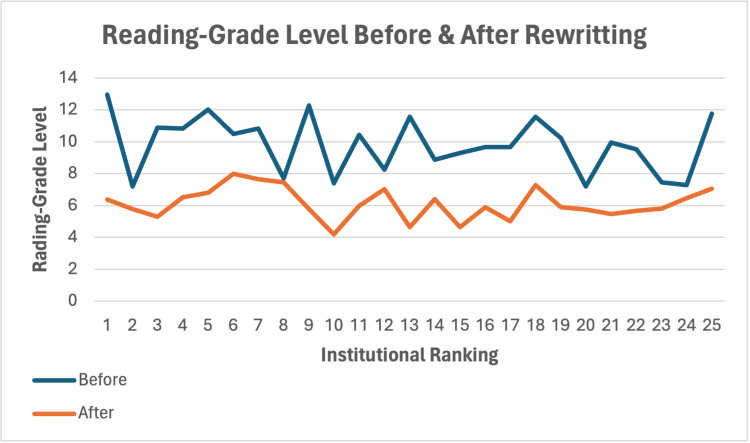
The average readability scores of patient education materials related to lateral epicondylitis from 25 of the top nationally ranked orthopaedic institutions, before and after generative AI-assisted editing. AI: artificial intelligence.

**Figure 2:**
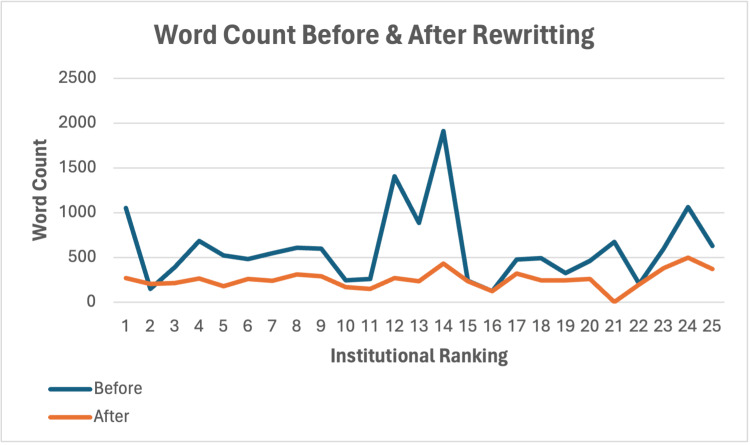
The average word count of patient education materials related to lateral epicondylitis from 25 of the top nationally ranked orthopaedic institutions, before and after generative AI-assisted editing. AI: artificial intelligence.

Twenty-two of the initial 25 orthopaedic institutions contained educational material related to LE on their websites. In the original, unedited PEM cohort, only six institutions obtained average readability scores below the eighth-grade reading level. The average reading grade level of all institutions’ original PEMs was 9.81 ± 1.76, and the average word count was 600.68 ± 409.44 words. Following ChatGPT’s edits to the original PEMs, all 25 rewritten PEMs obtained average readability scores below the eighth-grade reading level. The total average readability score for all rewritten PEMs was 6.12 ± 0.97 (Figure 1), with an average word count of 253.96 ± 100.76 (Figure 2). By utilising ChatGPT to rewrite the original PEMs, a reduction of 3.70 ± 1.84 (p<0.001) reading grade levels and 346.72 ± 364.63 (p<0.001) words was achieved (Table 2). The senior orthopaedic resident validated the accuracy of the information in the PEMs rewritten by ChatGPT.

**Table 2:** Results for paired t-test. Results for paired t-test to determine significance between (a) average readability scores and (b) total word count of the original and rewritten PEMs. PEM: patient educational material.

	Mean Reduction	Standard Deviation	95% Confidence Interval		p-value
			Lower	Upper	
Readability score	3.70	1.84	2.93	4.46	<0.001
Word count	346.72	364.63	196.21	497.24	<0.001

## Discussion

Our study examined patient educational material provided by 25 distinct orthopaedic institutions. Among them, 22 included PEMs pertaining to lateral epicondylitis. Only six of these institutions initially had PEM written at an eighth-grade reading level or lower. With minimal guidance, ChatGPT successfully revised the original PEMs, resulting in a decrease in average reading grade level and word count to meet the recommended guidelines by the CDC and NIH. These findings suggest that ChatGPT shows promise in enhancing the comprehensibility of PEMs for their target audience.

With the widespread availability of the internet at home, work, and while on the move, accessing PEMs have become more convenient than ever. Despite this improved access, the understanding of these materials has not seen a corresponding increase. Previous studies have shown a significant discrepancy between the CDC and NIH-recommended guidelines for writing PEMs and the true readability of PEMs published by healthcare institutions [18-20]. The implications of these studies are profound, as there is a well-documented correlation between low health literacy and worse clinical outcomes [1-3,21]. Moreover, a study investigating the impact of health literacy on patient satisfaction in surgical settings revealed that patients with higher health literacy tended to report greater satisfaction with their surgeries compared to those with lower health literacy level [22]. Health literacy has also been shown to be positively correlated with patient compliance [23]. Overall, PEMs that adhere to CDC and NIH guidelines not only improve patient outcomes, satisfaction, and compliance but also empower patients to make informed decisions about their healthcare. Artificial intelligence may be the solution to writing simpler and more effective PEMs.

In recent decades, there has been significant progress in artificial intelligence, with ChatGPT leading the way. These advancements have led to ChatGPT’s widespread integration across various sectors, including the medical field. A recent study on ChatGPT's ability to develop customized obesity treatment plans revealed its proficiency in crafting individualized strategies tailored to the specific requirements of each person [24]. Another study that looked at ChatGPT’s ability to manage obstructive sleep apnoea (OSA) determined that ChatGPT was able to demonstrate potential as a valuable resource for OSA diagnoses [25]. These studies showcase the multifaceted utility of ChatGPT within the healthcare sector, displaying its potential to revolutionise personalized treatment strategies for conditions like obesity and OSA. Building on this foundation, our study indicates that ChatGPT's capabilities extend further, demonstrating its capacity to craft PEMs that abide by the standards given by the CDC and NIH. This not a standalone finding. Previous studies have found that ChatGPT is not only capable of generating text-based information for specific audiences but is also preferred over content generated by humans [26-27]. This not only highlights ChatGPT's versatility but also underscores its pivotal role in enhancing patient comprehension and empowerment in medical decision-making processes [28-29].

There are a few noteworthy limitations to the present study. First, the CDC guidelines suggest considering demographic factors such as race, gender, and ethnicity when writing PEMs, which can be challenging when requesting assistance from a language processing model such as ChatGPT. Additionally, our evaluation of reading comprehension relied solely on reading grade-level comprehension scores and excluded videos, pictures, or other visual media that were included on institution’s websites. To our knowledge, there is no reliable metric that would have allowed us to objectively compare audiovisual media between hospital websites. Furthermore, ChatGPT is limited in its ability to incorporate, analyse, and regenerate pictures or diagrams, which was a critical aspect of our methodology. Ultimately, these visual tools may alter and potentially improve the viewer’s understanding of the information provided. Potential avenues for future research may include using AI to generate improved audiovisual multimedia in the healthcare space. Despite these limitations, our study is the first to successfully explore ChatGPT’s ability to generate PEMs that adhere to the CDC and NIH guidelines.

## Conclusions

Our study determined that ChatGPT was capable of rewriting PEM on LE at a reading comprehension level that abides by the CDC and NIH guidelines. Hospital administrators and orthopaedic surgeons should consider the findings of this study and the potential utility of artificial intelligence when crafting PEMs of their own.
